# Reliability of Heterochromatic Flicker Photometry in Measuring Macular Pigment Optical Density among Preadolescent Children

**DOI:** 10.3390/foods4040594

**Published:** 2015-10-16

**Authors:** Sasha M. McCorkle, Lauren B. Raine, Billy R. Hammond, Lisa Renzi-Hammond, Charles H. Hillman, Naiman A. Khan

**Affiliations:** 1 Division of Nutritional Sciences, University of Illinois, Louise Freer Hall, 906 S Goodwin Avenue, Urbana, IL 61801, USA; E-Mail: mccorkl2@illinois.edu; 2 Department of Kinesiology and Community Health, University of Illinois, Louise Freer Hall, 906 S Goodwin Avenue, Urbana, IL 61801, USA; E-Mails: lraine2@illinois.edu (L.B.R.); chhillma@illinois.edu (C.H.H.); 3 Department of Psychology, University of Georgia, 125 Baldwin Street, Athens, GA 30602, USA; E-Mails: bhammond@uga.edu (B.R.H.); lrenzi@uga.edu (L.R-H.)

**Keywords:** macular pigment optical density, heterochromatic flicker photometry, retinal lutein

## Abstract

Macular pigment optical density (MPOD)—assessed using customized heterochromatic flicker photometry (cHFP)—is related to better cognition and brain lutein among adults. However, the reliability of MPOD assessed by cHFP has not been investigated in children. We assessed inter-session reliability of MPOD using modified cHFP. 7–10-year-olds (*n* = 66) underwent cHFP over 2 visits using 11 examiners. Reliability was also assessed in a subsample (*n* = 46) with only 2 examiners. Among all participants, there was no significant difference between the two sessions (*p* = 0.59—session 1: 0.61 ± 0.28; session 2: 0.62 ± 0.27). There was no significant difference in the MPOD of boys *vs.* girls (*p* = 0.56). There was a significant correlation between sessions (Y = 0.52x + 0.31; *R*^2^ = 0.29, *p* ≤ 0.005), with a reliability of 0.70 (Cronbach’s α). Among the subsample with 2 examiners, there was a significant correlation between sessions (Y = 0.54x + 0.31; *R*^2^ = 0.32, *p* < 0.005), with a reliability of 0.72 (Cronbach’s α). In conclusion, there is moderate reliability for modified cHFP to measure MPOD in preadolescents. These findings provide support for future studies aiming to conduct noninvasive assessments of retinal xanthophylls and study their association with cognition during childhood.

## 1. Introduction

Lutein is a plant pigment that has been shown to preferentially accumulate in the macula of the neural retina as macular pigment (MP) and across all brain cortices of infants and adults [1,2]. Macular pigment optical density (MPOD), comprised of lutein, zeaxanthin, and meso-zeaxanthin, has historically been strongly linked to preventing age-related macular degeneration [3], but converging evidence now also points to a positive role of MPOD in neurocognition [4,5]. Among non-human primates, MPOD has been found to serve as a good proxy for the amount of lutein and zeaxanthin in the brain [6]. Higher MPOD has also been associated with faster visual processing speed [7] and cognitive performance among adults [4,8,9]. Therefore, MPOD may be used as a biomarker of tissue levels of carotenoids with neurocognitive potential.

MPOD assessments, however, in pediatric populations are limited. This is a concern because recent work demonstrates that lutein’s status as the predominant carotenoid in the human brain is evident in early life [1]. The scarcity of MPOD data in children has resulted in limited knowledge of the importance of dietary or brain lutein for optimal cognitive function and brain development [10,11]. One of the challenges in accumulating these valuable data is that heterochromatic flicker photometry (HFP), the widely used non-invasive technique of assessing MPOD in adults, has unknown reliability in preadolescent children. HFP exploits the spectral absorption properties and retinal location of macular pigment by presenting a light stimulus of two alternating wavelengths at the fovea and at a parafoveal location. Thus, successful completion of HFP requires participant compliance and understanding, which is likely dependent on age, to determine their respective null flicker zones. The ability to measure MPOD in children would be beneficial for studies looking to investigate how lutein and zeaxanthin relate to childhood cognitive function and development. This is particularly important given that the correlations between measurements of dietary and serum lutein and zeaxanthin and retinal concentrations are weak [12]. Further, this noninvasive technique would be particularly suitable for pediatric populations that may be reluctant to undergo venous blood draws for biomarker assessment.

Previous studies have provided extensive support for the reliability of HFP among adult populations [13]. Studies to date have shown that correlation coefficients across two HFP sessions on non-consecutive days vary between 0.58–0.97 [14,15,16]. Interestingly, some studies have shown sex differences in MPOD such that males have up to 38% higher MPOD than females despite having similar plasma carotenoid concentrations [17]. Another study observed differences across sex based on age such that men between 40 to 49 years had higher MPOD than women, while women between 50 to 59 years had higher MPOD than men [18]. On the other hand, other studies have shown no difference in MPOD by sex [19,20]. Whether or not these differences across sex are evident in childhood remains unknown due to the lack of MPOD data among pediatric populations.

Assessing the reliability of HFP is the necessary first step in establishing its use for measuring MPOD in child populations. Accordingly, the major aim of the current study was to determine the test–retest reliability of HFP among a group of healthy male and female preadolescent children. Secondary aims of the study were to: (1) delineate the effects of minimizing the number of examiners on HFP reliability; and (2) assess whether differences in MPOD across sex are evident in preadolescence. Our primary hypothesis was that HFP would be moderately reliable among preadolescent children over two non-consecutive sessions. Further, we hypothesized that HFP reliability will be higher among a subsample of participants tested by two examiners and that sex differences in MPOD will be observed among preadolescent children.

## 2. Experimental Section

### 2.1. Participants

Preadolescent children (*n* = 66) between the ages of 7 and 10 years from the East-Central Illinois community were recruited to participate in this study. Exclusion criteria included presence of neurological disorders, physical disabilities, and psychoactive medication status. Additionally, all participants had normal or corrected-to-normal vision. All participants provided written assent and their legal guardians provided written informed consent. All procedures were in accordance with the ethical standards and regulations of the University of Illinois at Urbana-Champaign (Institutional Review Board number 12321).

### 2.2. MPOD Measurement

MPOD was measured using customized HFP (cHFP) by a macular densitometer (Macular Metrics Corporation, Rehoboth, MA, USA) that was identical to a version described by Wooten *et al.* [21] except that it did not allow for an assessment of the entire spatial profile. This procedure has been described previously [22]. This study, however, utilized a slightly varied form of the procedure typically described in adult studies. Among unimpaired adults, participants manipulate the radiance of the short-wave component of the test stimulus themselves (method of adjustment) to produce a null flicker zone, after receiving instruction from a trained experimenter. In children, the psychophysical technique was modified as described previously by Renzi *et al.* [9] for older adults with mild cognitive impairment. Briefly, instead of using the method of adjustment to find thresholds, the examiner manipulated the radiance of the short-wave component of the test stimulus, using simplified instructions (method of limits). After the null zone was found via the method of limits, the method of constant stimuli was used to further narrow the range of the null zone.

### 2.3. Training of Examiners

Among all participants (*n* = 66), 20 had two different examiners at the two time points (Subgroup 1) while the remaining 46 participants had the same examiner at both time points (Subgroup 2). Additionally, in Subgroup 1 there were 11 different examiners while in Subgroup 2 there were only 2 examiners. All examiners were initially trained by the same person and were required to serve as a participant prior to being an examiner themselves. Subsequently, examiners from Subgroup 1 tested at least two other people to ensure they were comfortable with running the device. The examiners in Subgroup 2 underwent more extensive training. Specifically, examiners for Subgroup 2 tested 15 adults on two occasions to ensure they were able to replicate adult reliability data currently in the literature. After successful completion of this training protocol they then proceeded to test the children in this study.

### 2.4. Statistical Analysis

Paired *t* tests were conducted among the entire sample as well as the two subgroups separated by number of examiners (*i.e.*, 11 examiners *vs.* 2 examiners). In addition, an independent *t* test was conducted to determine differences between boys and girls. Next, bivariate correlations between the sessions were conducted. The intersession reliability was tested with Cronbach’s α. All statistical calculations were performed in SPSS (IBM SPSS Statistics, Version 22.0). The α level was set at 0.05 and was based on a one-tailed criterion for significance. Additionally, Bland–Altman plots were constructed to examine if there was any bias between the sessions.

## 3. Results

Among all participants (*n* = 66), 20 had two different examiners at the two time points (Subgroup 1) while the remaining 46 participants had the same examiner at both time points (Subgroup 2). For 34 of these participants, time to complete the task was recorded. For this subset of participants on average it took 11.38 ± 2.63 min to complete this task. Additionally, for these 34 participants the dates between testing were 10.24 ± 9.48 days. Table 1 describes the sex distribution and ages of the different groups.

**Table 1:** Participant characteristics separated by group.

Characteristic	Full Sample (*n* = 66)	Subgroup 1 (*n* = 20)	Subgroup 2 (*n* = 46)
Sex [*n* (%)]			
Male	26 (39)	14 (70)	12 (26)
Female	40 (60)	6 (30)	34 (74)
Age (years)	8.8 ± 0.08	8.7 ± 0.13	8.8 ± 0.11

**Figure 1:**
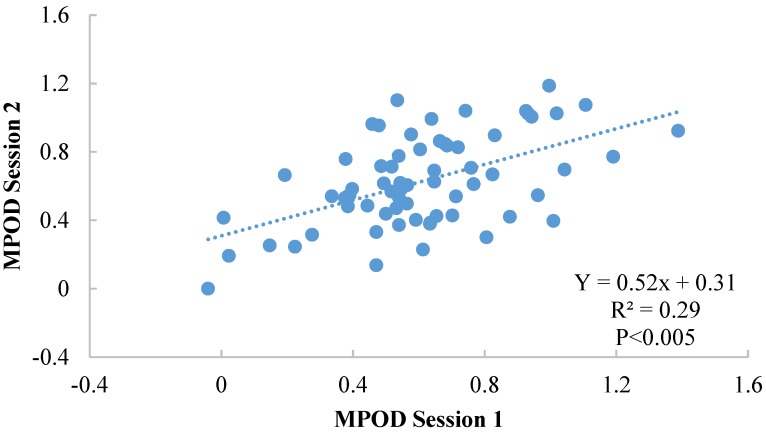
Illustration of the correlation between sessions 1 and 2 for the full sample (*n* = 66).

Among the full sample, there was no significant difference between MPOD at the two sessions (*p* = 0.59—session 1: 0.61 ± 0.28; session 2: 0.63 ± 0.27) and no significant difference was found between boys and girls (*p* = 0.56). Furthermore, there was a significant correlation between sessions (Y = 0.52x + 0.31 (see Figure 1); *r* = 0.54, *p* < 0.005), with an intersession reliability of 0.70 (Cronbach’s α).

**Figure 2:**
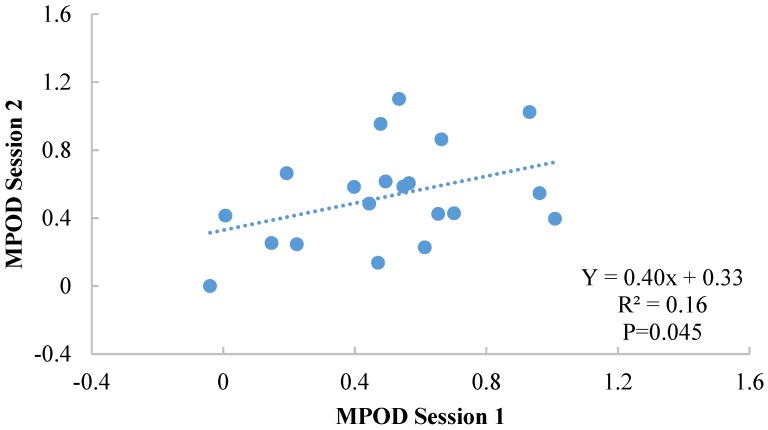
Illustration of the correlation between sessions 1 and 2 for Subgroup 1 (11 examiners).

Results among Subgroup 1 are illustrated in Figure 2. There was no significant difference between the two sessions (*p* = 0.71—session 1: 0.50 ± 0.29; session 2: 0.53 ± 0.29) and no significant difference was found between boys and girls (*p* = 0.53). However, in this subgroup the correlation between the two sessions was reduced (Y = 0.4x + 0.33; *r* = 0.39, *p* = 0.045; based on a one sample test), and the intersession reliability was 0.57 (Cronbach’s α).

**Figure 3:**
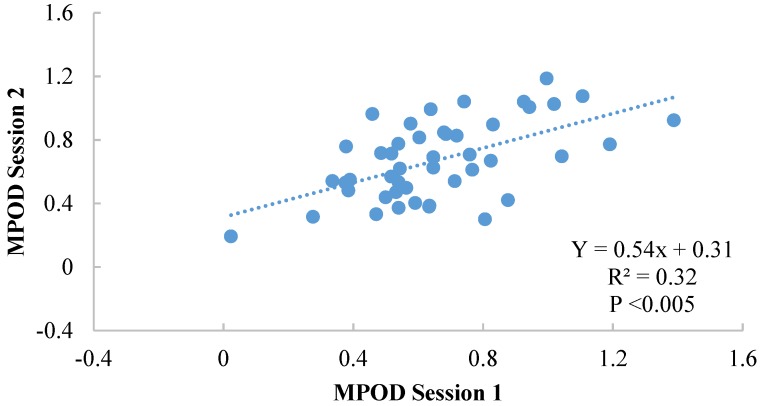
Illustration of the intersession correlation between sessions 1 and 2 for Subgroup 2 (2 examiners).

Results for Subgroup 2 are illustrated in Figure 3. There was no significant difference between the two sessions (*p* = 0.71—session 1: 0.66 ± 0.26, session 2: 0.67 ± 0.24) and no significant difference was found between boys and girls (*p* = 0.29). Moreover, there was a significant correlation between sessions (Y = 0.54x + 0.31; *r* = 0.57, *p* <0.005), with an intersession reliability of 0.72 (Cronbach’s α).

**Figure 4:**
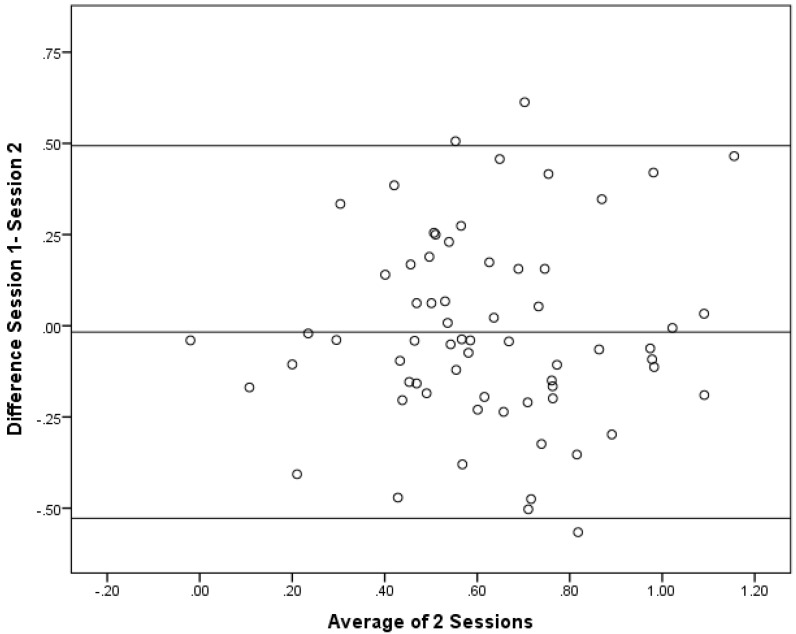
Bland–Altman Plot for the Full sample (*n* = 66).

**Figure 5:**
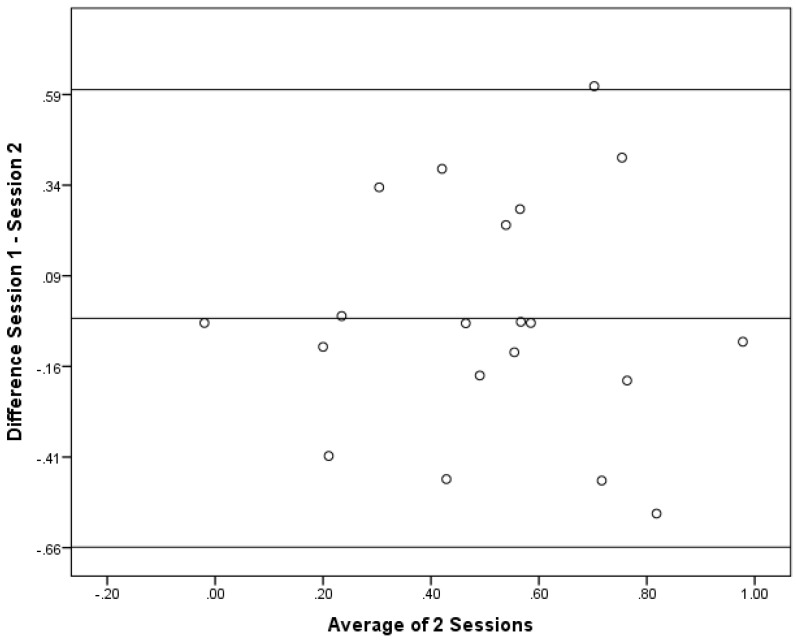
Bland–Altman Plot for the Subgroup 1 (*n* = 20).

**Figure 6:**
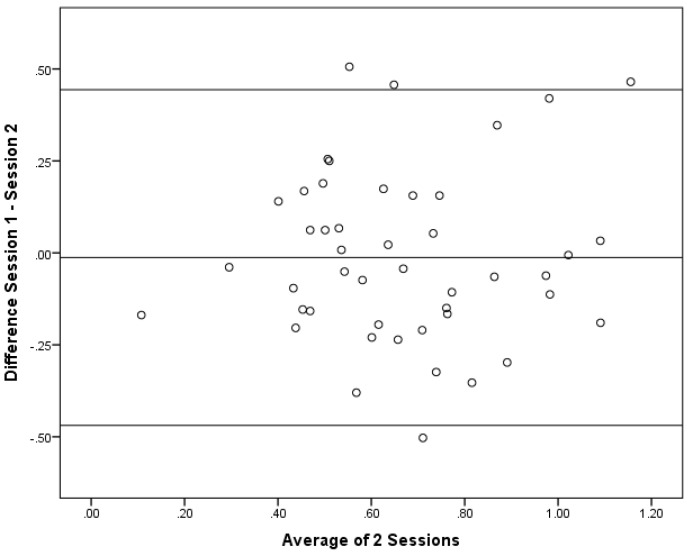
Bland–Altman Plot for Subgroup 2 (*n* = 46).

As correlations do not show if the there is a bias between the sessions, Bland–Altman plots were constructed. Bland–Altman plots are a simple way to evaluate a bias between mean differences, and to estimate the agreement interval, which is where 95% of the differences of the second session, compared to the first session, fall. These plots are illustrated in Figure 4, Figure 5 and Figure 6.

When performing a one-sample *t* test, none of the groups had a mean difference that was significantly different from 0. The results from the one-sample *t* test were: the full sample (*p* = 0.59); Subgroup 1 (*p* = 0.71); and Subgroup 2 (*p* = 0.71). This result indcates that there was general agreement between the sessions. In general, these data demonstrate that there was no directional bias beteween the sessions as the differences at different magnitudes of the measurement remain constant. Since this was conducted as a *post-hoc* comparison, there was no *a priroi* hypothesis for the maximum acceptable differences. Thus, firm conclusions cannot be drawn regarding the acceptablity of the differences. The largest 95% confidence intervals, however, were in Subgroup 1 with an upper limit of 0.60 and a lower limit of −0.66. For the full sample, the upper and lower limits were 0.49 and −0.53, whereas Subgroup 2 had the most narrow upper and lower limits of 0.44 and −0.47.

## 4. Discussion

Our results indicate that HFP is a moderately reliable technique for the assessment of MPOD in preadolescent children. Among the full sample of participants, we observed a Cronbach alpha value of 0.70 between the two sessions. This level of intersession reliability is similar to that observed among adult studies that reported on test–retest of five sessions with a Cronbach alpha of 0.68 [23]. However, this level of reliability is considerably lower when compared to other studies reporting intersession correlations over ten sessions (Cronbach alphas of 0.89 and 0.97) [21,24]. Further, the results from this study revealed that limiting the examiners improves on the reliability of the measurement in children from low to moderate (Cronbach α’s 0.57 and 0.72 respectively). This improvement in reliability certainly suggests the role of the examiner is important in obtaining reliable MP data when measuring groups such as children or cognitively impaired adults. In this study, a method of limits and constant stimuli was used instead of the traditional adjustment method often used in HFP studies with normal adults. Refining this procedure even further could possibly lead to even better reliability with groups such as young children.

The results of the current study are within the range seen in previous data collected from adult and child cohorts. The correlation coefficients obtained in this study, 0.54, 0.39, and 0.57 are marginally lower than in adult studies that tested at two time points 0.58–0.97 [14,15,16], but are not far from the lower spectrum of this range. These test–retest correlations demonstrate that children can perform similarly across two time points (e.g., average MPOD across the two sessions was the same). Regarding the raw MPOD data, the MPOD values obtained in our study are similar to a previous sample of 6–12-year-old Chinese children (MPOD 0.56 ± 0.25). That study also did not find a significant difference in MPOD between males and females [25]. Nevertheless, the MPOD values obtained in our sample of children appears to be considerably higher than what is typically observed in many adult Western populations (mean range between 0.21 and 0.48) [18,20,24,26]. Higher levels in this sample may be due simply to our sampling, which might not adequately reflect the generally low carotenoid intake of many children in the USA [27].

Some previous studies among adults have observed sex differences in MPOD. Therefore, the finding that there were no significant differences between boys and girls in the present study was contrary to our *a priori* hypothesis. However, these results are not entirely surprising given that differences across sex are not consistent among adult samples. Further, the sex differences in MPOD have been shown to be mediated by age among adults. The implication of our findings is that, if sex differences in MPOD truly exist among adults it is likely that these differences manifest following adolescence (possibly suggesting a hormonal influence; e.g., [28]).

The moderate level of reliability for HFP observed here provides preliminary support for future studies aiming to study associations between MPOD and cognitive function and brain health. The HFP technique has several advantages to testing this noninvasive *in vivo* technique in children compared with other techniques available for measuring MPOD. First, the participants may choose which eye to use for the test as no difference has been found in the macular pigment between the left and right eyes [29]. This particular test is convenient for the use in children due to not needing to dilate the pupils, which is necessary in other tests [21]. Another benefit of using this measurement in children is that head movement does not impact the MPOD value while other techniques, including the Maxwellian view measurement, utilize a dental impression bite bar and headrest assembly to stabilize the head [21]. Finally, it reflects actual levels of these food components in neural tissue (noninvasive assessment allowing interventions to be followed). Assessments in serum and diet are only weakly correlated to levels of xanthophylls in the central nervous system.

A limitation of this study is that it investigated only test–retest reliability. This study does not evaluate the validity of measuring MPOD by HFP in children. Without validation data we are unable to clarify whether the consistency between the two sessions was due to the validity of the HFP technique or a consequence of poor comprehension of task instruction at the two sessions.

Since subjects vary greatly in MPOD [30,31], it is important to know whether or not measurements can be made reliably in neural tissues [6]. Dietary lutein and zeaxanthin, the carotenoids composing the MP [32], can be measured by food frequency questionnaires, food records and food recalls. While the relationship between dietary intake and MPOD was found to be significant and positive in three studies, this result is not consistent in the literature [12]. Thus the use of HFP to measure MPOD in preadolescent children will serve future studies that may look to investigate the relationship between cognitive functioning and levels of lutein measured directly in central nervous system tissue (the retina).

## 5. Conclusions

Given that HFP is a subjective technique that requires a response from the participant, it is important to test whether preadolescent children can reliably make perceptual judgments about the presence or absence of flicker before relating it to other measures. The results from the current study demonstrate that using HFP to measure MPOD is a moderately reliable tool in children. Future studies should limit the examiners, as this was found to improve the reliability of the task in children. Further, researchers would be well-advised to consider variations in the procedure that could further improve the reliability of this technique. This will be a benefit to future studies that look to examine how macular pigment density, a surrogate measure of brain levels of xanthophylls, particularly lutein [6], relates to other dependent measures in children. The finding that male and female children have similar MPOD levels suggests that children can be considered collectively without the need for analysis by sex.
